# Direct Conversion of Human Stem Cell-Derived Glial Progenitor Cells into GABAergic Interneurons

**DOI:** 10.3390/cells9112451

**Published:** 2020-11-10

**Authors:** Jessica Giacomoni, Andreas Bruzelius, Christina-Anastasia Stamouli, Daniella Rylander Ottosson

**Affiliations:** 1 Group of Developmental and Regenerative Neurobiology, Wallenberg Neuroscience Center and Lund Stem Cell Center, Department of Experimental Medical Science, Faculty of Medicine, Lund University, 221 84 Lund, Sweden; jessica.giacomoni@med.lu.se; 2 Group of Regenerative Neurophysiology, Lund Stem Cell Center, Department of Experimental Medical Science, Faculty of Medicine, Lund University, 221 84 Lund, Sweden; andreas.bruzelius@med.lu.se (A.B.); ch4247st-s@student.lu.se (C.-A.S.)

**Keywords:** human embryonic stem cells, neurological disorders, PDGFRα, GFAP, cellular reprogramming, induced neurons, direct conversion, PV, patch-clamp electrophysiology

## Abstract

Glial progenitor cells are widely distributed in brain parenchyma and represent a suitable target for future therapeutic interventions that generate new neurons via in situ reprogramming. Previous studies have shown successful reprogramming of mouse glia into neurons whereas the conversion of human glial cells remains challenging due to the limited accessibility of human brain tissue. Here, we have used a recently developed stem cell-based model of human glia progenitor cells (hGPCs) for direct neural reprogramming by overexpressing a set of transcription factors involved in GABAergic interneuron fate specification. GABAergic interneurons play a key role in balancing excitatory and inhibitory neural circuitry in the brain and loss or dysfunction of these have been implicated in several neurological disorders such as epilepsy, schizophrenia, and autism. Our results demonstrate that hGPCs successfully convert into functional induced neurons with postsynaptic activity within a month. The induced neurons have properties of GABAergic neurons, express subtype-specific interneuron markers (e.g. parvalbumin) and exhibit a complex neuronal morphology with extensive dendritic trees. The possibility of inducing GABAergic interneurons from a renewable in vitro hGPC system could provide a foundation for the development of therapies for interneuron pathologies.

## 1. Introduction

Direct neuronal reprogramming is a rapidly emerging field of research where non-neuronal cells can be converted into induced neurons (iNs) using key combinations of transcription factors, microRNA, and/or a cocktail of chemical compounds that manipulate pathways involved in neuronal fate and functions. Considerable progress has been made since the first demonstration of human iNs [1,2] and nowadays, the possibility of obtaining different subtype-specific iNs hold promise for many biomedical applications. The advantages include the generation of patient- and disease-specific neurons for disease modeling and drug screening [3,4], and the establishment of a renewable source of neurons for cell-replacement therapies. Direct conversion bypasses the pluripotent stage and thus provides a faster and potentially safer way of obtaining neurons in vitro for autologous and allogenic cell transplantation [5,6]. The direct conversion also allows for the generation of functional iNs directly in the brain by reprogramming resident cells and thus represents a route for in situ brain repair [7].

Glial progenitor cells (GPCs), also known as oligodendrocyte progenitor cells (OPCs) or NG2 cells, represent a particular suitable target for direct in vivo reprogramming due to their proliferative nature and ubiquitous distribution in the brain parenchyma [8,9]. In addition, GPCs are equipped with a necessary machinery of post-synaptic compartments, potentially facilitating the synaptic integration of the glia-derived iNs [10]. As a proof-of-principle, several labs have thus far successfully converted endogenous glia into functional neurons within the rodent brain [11,12,13,14,15]. 

We have previously reported that GABAergic interneurons of the parvalbumin (PV) subtype can be generated from NG2 resident glial cells in the mouse brain [16,17]. GABAergic interneurons have an essential role in balancing the excitatory and inhibitory neural circuitry in the nervous system and include many different subtypes that can be distinguished by specific gene expression profiles, morphology, and function [18,19,20]. Among the various subtypes, PV interneurons have been specifically implicated in epilepsy and cognitive impairments (e.g. schizophrenia and autism) with subtle pathologies such as decreased neurite number and diminished neural connectivity [21,22]. Recent studies have suggested the possibility of tackling several of these diseases with the transplantation of GABAergic interneurons [21,23]. However, the ability to induce subtype-specific interneurons via in situ direct reprogramming may represent an alternative therapeutic approach to restore the lost neuronal function in the brain. In this regard, the conversion of human cells into clinically relevant interneurons provides an important step toward developing a new cell repair strategy. However, derivation of GPCs from human fetal brain tissue has proved to be challenging, mainly due to the difficulty in sourcing them as they arise late in the second trimester [24].

In this study, we used a recently developed human embryonic stem cell (hESC)-derived model of GPCs [25] for neural reprogramming with the aim to generate GABAergic interneurons. We used a combination of transcription factors that has previously been shown to generate GABAergic interneurons including PV interneurons from other cell sources [26] and compare it with or without the addition of RE1-silencing transcription factor (REST) inhibition, known to improve the conversion of human somatic cells [3,27]. Our results show that hGPCs are able to convert into functional neurons in only 26 days. The neurons develop a mature neuronal morphology and a GABAergic phenotype that was characterized by the identification of relevant markers such as PV and γ-aminobutyric acid (GABA) as well as GABAergic synaptic network activity. The induced cultures consisted of a heterogenous population of several interneuron subtypes and a low proportion of non-GABAergic cells.

This is the first successful conversion of hGPCs into functional and subtype-specific GABAergic neurons that might not only have potential for cell-restorative therapies, but also for disease modeling strategies. 

## 2. Materials and Methods

### 2.1. hESC-Derived GPC Culturing

GPCs were generated from two different hESC-lines: RC17 (RCe021-A, p26–30) and HS1001 (Kle055-A, p28). The hESCs were expanded in IPS-Brew XF medium (StemMACS, Miltenyi, Bergisch Gladbach, Germany) on LN521 (0.5 µg/cm^2^, Biolamina, Sundbyberg, Sweden) coated tissue plates at 37 °C in 5% CO_2_ and passaged weekly with ethylenediaminetetraacetic acid (EDTA, 0.5 mM, Gibco, Thermo Fisher; Waltham, MA, USA). For details on the glial differentiation protocol and cryopreservation, see Nolbrant et al. [28].

### 2.2. Flow Cytometry Analysis

hESC-derived GPCs between day 188 and 304 were dissociated for 8 min in Accutase (StemPro, Thermo Fisher, Waltham, MA, USA) and resuspended to 1 million cell/mL in Miltenyi wash buffer (phosphate-buffered saline [PBS, Gibco, Thermo Fisher; Waltham, MA, USA] + 0.5% bovine serum albumin Fraction V [BSA, Gibco, Thermo Fisher, Waltham, MA, USA] + 2 μM EDTA + 0.05‰ Phenol Red [Sigma-Aldrich, St. Louis, MO, USA]). A total of 100 μL of cell suspension was aliquoted per flow cytometry tube and incubation with fluorochrome-labeled antibodies (PE anti-human CD140a, 1:10 [BD Biosciences, cat. no. 556002, Eysins, Switzerland]; APC anti-CD44, 1:500 [Miltenyi, cat. no. 130-095-177, Bergisch Gladbach, Germany]; APC anti-human CD133/1, 1:50 [Miltenyi, cat. no. 130-113-668, Bergisch Gladbach, Germany]; FITC anti-human SSEA-4, 1:20 [Biolegend, cat. no. 330410, San Diego, CA, USA] was performed for 15 min at 4 °C. Cells were washed once using Miltenyi wash buffer, centrifuged for 10 min at 200× *g*, and transferred to 5 mL polystyrene tubes with cell-strainer caps in DMEM/F12 (Gibco, Thermo Fisher, Waltham, MA, USA) + DNAse (Sigma-Aldrich, St. Louis, MO, USA). To exclude dead cells from the analysis, propidium iodide (PI, 1:500, Miltenyi, cat. no. 130-095-177, Bergisch Gladbach, Germany) was added to the samples. For each sample, 10,000 events were analyzed on a FACSAria III sorter (BD Biosciences, Eysins, Switzerland). Gates were set based on Fluorescence Minus One (FMO) controls and compensation was performed using single-stained cells. Flow cytometry data were analyzed using the software FlowJo 10.7 (BD Biosciences, Eysins, Switzerland).

### 2.3. Viral Vectors

As two of the transcription factors used in this study elicit the initial phase of the cell fate changes and require downregulation at a defined time [26], we used a mix of constitutive promoter guided- and inducible promoter guided-transcription factors. The non-regulated system consisted of third-generation lentiviral vectors (LVs) expressing open reading frames (ORFs) for Ascl1 (mouse), Dlx5 (human), and Lhx6 (human) under the phosphoglycerate kinase (PGK) promoter for ubiquitous expression while the doxycycline-regulated system (TET-ON promoter) included LVs expressing ORFs for Sox2 (mouse) and Foxg1 (human) always co-transduced with a TET-ON trans-activator (FUW.rtTA-SM2, Addgene, Watertown, MA, USA). Two shRNAs targeting REST were expressed in lentiviral constructs under the control of a non-regulated U6 promoter. For monitoring the neuronal maturation and labeling cells in whole cell patch-clamp recordings, we used a lentivirus expressing red fluorescent protein (RFP) under the control of the human Synapsin 1 promoter (hSyn-RFP; Addgene, Watertown, MA, USA). Doxycycline-regulated LVs expressing mouse Ascl1, Lmx1a, and Nurr1 cDNAs have been previously described [28]. LVs were produced as previously described [29] and titrated by quantitative PCR analysis [30]. All the titers were in a range of 6 × 10^8^ and 6 × 10^9^ transduction units/mL.

### 2.4. Neural Direct Conversion

hESC-derived GPCs were dissociated at single cells using Accutase (StemPro, Thermo Fisher; Waltham, MA, USA) and seeded in a glial medium at a density of 50,000 cells/cm^2^ either on 24-well plates (for ICC and RNA extraction) or on glass coverslips in 24-well plates (for electrophysiology) that had been pre-treated according to Richner et al. [31]. Prior to seeding, wells and coverslips were serially coated with polyornithine (15 μg/mL, Sigma-Aldrich, St. Louis, MO, USA), laminin (5 μg/mL; Thermo Fisher, Waltham, MA, USA), and fibronectin (0.5 ng/μL, Thermo Fisher, Waltham, MA, USA). Lentiviral transduction was performed 24 h after plating and occasionally after 72 h based on cell recovery from seeding, at a multiplicity of infection (MOI) of 1–2 per vector. The following day, the culture medium was completely changed, and the inducible transgene expression allowed upon doxycycline (2 μg/mL, Duchefa, Haarlem, Netherlands) addition. 72 h after transduction, medium was replaced by neural differentiation medium (NDiff227, Takara-Clontech, Gothenburg, Sweden) containing doxycycline (2 μg/mL), the small molecules (SMs): CHIR99021 (2 μM, Axon, Groningen, Netherlands), SB-431542 (10 μM, Axon, Groningen, Netherlands), noggin (0.5 μg/mL, R&D Systems, Minneapolis, MN, USA), LDN-193189 (0.5 μM, Axon, Groningen, Netherlands), and valproic acid sodium salt (VPA; 1 mM, Merck Millipore, Burlington, MA, USA); and the growth factors (GFs): LM-22A4 (2 μM, R&D Systems, Minneapolis, MN, USA), GDNF (2 ng/mL, R&D Systems, Minneapolis, MN, USA), NT3 (10 ng/mL, R&D Systems, Minneapolis, MN, USA), and db-cAMP (0.5 mM, Sigma-Aldrich, St. Louis, MO, USA). Two-thirds of the medium was refreshed every 2–3 days. At 12–14 days post transgene activation, doxycycline and SMs were withdrawn from the neuronal differentiation medium and the reprogrammed cells were further on cultured using medium supplemented with only GFs to promote neuronal maturation. At the same time, cells for electrophysiology were transduced with hSyn-RFP lentivirus at MOI of 1–2 and the media changed the day after.

### 2.5. Reverse Transcription-Quantitative Polymerase Chain Reaction (RT-qPCR)

Total RNA was extracted with the RNeasy Micro Kit (Qiagen, Hilden, Germany) and 150–300 ng RNA from each sample reverse-transcribed using a Maxima First Strand cDNA Synthesis Kit (Thermo Fisher, Waltham, MA, USA). The Bravo Automated Liquid Handling Platform (Agilent, Santa Clara, CA, USA) was used to pre-mix cDNA (1 μL), LightCycler 480 SYBR Green I Master (5 μL, Roche, Basel, Switzerland), and relevant primers (4 μL, see Table S1; Integrated DNA Technologies [Coralville, IA, USA] and Invitrogen [Carlsbad, CA, USA]) in 384-wells subsequently analyzed by quantitative RT-PCR on a LightCycler 480 II instrument (Roche, Basel, Switzerland) using a 40 cycles, two-step protocol (95 °C, 30-s denaturation and 60 °C, 1-min annealing/elongation). For each sample, the relative gene expression was calculated from technical triplicates using the ∆∆CT method in which the day −1 of the reprogramming (starting cells before conversion was initiated) was set as the control. qPCR data were normalized against two housekeeping genes (*GAPDH* and *ACTB*, see Table S1).

### 2.6. Immunocytochemistry and Microscopy

Prior to staining, the cells were fixed in 4% paraformaldehyde (with the addition of 0.1% glutaraldehyde (cat. no. G6257, Sigma-Aldrich, St. Louis, MO, USA) for GABA staining for 15 minutes at room temperature. Cells were washed three times with PBS, pre-incubated in blocking solution (0.1% Triton X-100 [Sigma-Aldrich, St. Louis, MO, USA] + 5 % donkey serum + PBS) for 1–3 hours and then incubated with the primary antibodies (listed in Table S2) in blocking solution over night at 4 °C. The following day, cells were washed twice with PBS and incubated in blocking solution for 30 min before adding the secondary fluorophore-conjugated antibodies (1:200, Jackson ImmunoResearch Laboratories, West Grove, PA, USA) and DAPI (1:500, Sigma-Aldrich, St. Louis, MO, USA) in blocking solution for 1 h at room temperature. Finally, cells were thoroughly washed with PBS. Fluorescent images were captured using either a Leica DMI6000B widefield microscope or a Leica TCS SP8 confocal laser scanning microscope (Figure S1) and acquired using the Leica LAS X software (Leica, Wetzlar, Germany). The images were processed using Adobe Photoshop 2020 (Adobe, San Jose, CA, USA) and the adjustments were applied equally across the entire image, and without loss of information.

### 2.7. High-Content Screening

The neuronal purity was calculated as the number of Cy3-Tau^+^ cells over the DAPI^+^ nuclei using a Cellomics Array Scan VTI HSC reader and the HCS Studio 3.0 Scan software (Thermo Fischer, Waltham, MA, USA), which is an automated method ensuring reproducibility and reliability in quantitative imaging. For each experiment, the “Target Activation” program was applied to acquire 198 fields (10X magnification) from a well, where the accurate measurement of the average fluorescence for antigen-positive cells was defined above the background fluorescence of antigen-negative cells (internal negative control). The same array was used for the analysis of the number of Cy2-PV^+^ or Cy2-CALB1^+^ cells containing DAPI^+^ nuclei and expressing Cy3-Tau and for the quantification of GABAergic neuronal purity calculated as the number of Cy3-GABA^+^ cells containing DAPI^+^ nuclei and expressing Cy2-Tau. The neurites of Cy2-Tau^+^/DAPI^+^ and Cy2-PV^+^/Cy3-Tau^+^/DAPI^+^ cells were analyzed in number, length, and PV expression by applying the program “Neuronal Profiling”. 

### 2.8. Electrophysiology

Whole cell patch-clamp electrophysiological recordings were performed at four weeks and eight weeks post-conversion. Cells were cultured on glass coverslips and transferred to a recording chamber with a constant flow of Krebs solution gassed with 95% O_2_–5% CO_2_ at room temperature. The composition of the Krebs solution was (in mM): 119 NaCl, 2.5 KCl, 1.3 MgSO_4_, 2.5 CaCl_2_, 25 Glucose and 26 NaHCO_3_. For recordings, a Multiclamp 700B amplifier (Molecular Devices, San Jose, CA, USA) was used together with borosilicate glass pipettes (3–7 MOhm) filled with the following intracellular solution (in mM): 122.5 potassium gluconate, 12.5 KCl, 0.2 EGTA, 10 Hepes, 2 MgATP, 0.3 Na_3_GTP, and 8 NaCl adjusted to pH 7.3 with KOH as in [2]. Data acquisition was performed with pClamp 10.2 (Molecular Devices, San Jose, CA, USA); the current was filtered at 0.1 kHz and digitized at 2 kHz. One week prior to recording, cells were transduced with a lentivirus for hSyn-RFP. The cells for recordings were selected based on expression of RFP, a clean surface, and neuronal morphology. Directly after break in resting, membrane potential was immediately noted in current clamp mode, thereafter, cells were kept at a membrane potential of −60 mV to −70 mV and 500 ms currents were injected from −20 pA to +35 pA with 5 pA increments to induce action potentials. For inward sodium and delayed rectifying potassium current measurements, cells were clamped at −70 mV and voltage-depolarizing steps were delivered for 100 ms at 10 mV increments. Spontaneous activity was recorded in voltage-clamp mode at −70 mV. Cyanquixaline (CNQX, Tocris, Bristol, United Kingdom) and picrotoxin (PTX, Tocris, Bristol, United Kingdom) were added to the Krebs solution at a final concentration of 50 and 100 µM, respectively, to block α-amino-3-hydroxy-5-methyl-4-isoxazolepropionic acid (AMPA) and GABA_A_ receptors in a few selected cells. The properties of single action potentials were measured from the first observed spike evoked by the rheobase current injection step. Sodium and potassium channels were selectively blocked using 1 µM tetrodotoxin (TTX, Tocris, Bristol, United Kingdom) and 10 mM tetraethyl ammonium (TEA, Tocris, Bristol, United Kingdom). Data were analyzed using the software Clampfit 10.3 (Molecular Devices, San Jose, CA, USA) and Igor Pro 8.04 (Wavemetrics, Portland, OA, USA) combined with the NeuroMatic package [32].

### 2.9. Statistical Analysis

Normality was assessed using the Shapiro–Wilk test and outliers were identified using Grubbs’s test and removed from the dataset. For gene expression and high content screening, data were analyzed using a two-tailed unpaired t-test (with Welch´s correction in the case of unequal variance assessed by the F test) when comparing two groups and the Kruskal–Wallis test followed by Dunn’s post-hoc test for multiple comparisons of reprogramming and neural conversion media (NDiff) groups with the glial medium control (GM). n represents replicates from independent experiments. For physiological properties, data were analyzed using two-tailed unpaired Mann–Whitney for all comparisons except for postsynaptic activity with antagonist (CNQX), where paired Wilcoxon rank-sum tests were performed. n represents the number of cells successfully patched. All data are represented either as means or means ± SEM. * *p* < 0.05; ** *p* < 0.01; *** *p* < 0.001; **** *p* < 0.0001. Statistical analyses were conducted using GraphPad Prism 8.4.2 (GraphPad, San Diego, CA, USA).

## 3. Results

### 3.1. Five Factor Combination Converts hESC-Derived GPCs into Induced Neurons

In an initial series of experiments, we examined whether hGPCs from a recently developed pluripotent stem cell-based model [25] could be reprogrammed into neurons with a combination of five transcription factors that have previously been proven to successfully convert mouse and human fibroblast into induced GABAergic telencephalic neurons [26]. Prior to reprogramming, we assessed the phenotype of the glial population by fluorescent activated cell sorting (FACS), which confirmed the presence of three subtypes of glial progenitors (Table S3): primarily oligodendrocyte-biased (58.8 ± 2.7% CD140^+^/CD44^−^, n = 12), a minority of bipotent (14.8 ± 2.5% CD140^+^/CD44^+^, n = 12), and astrocyte-biased (17.9 ± 3.1% CD44^+^/CD140^−^, n = 12).

**Figure 1:**
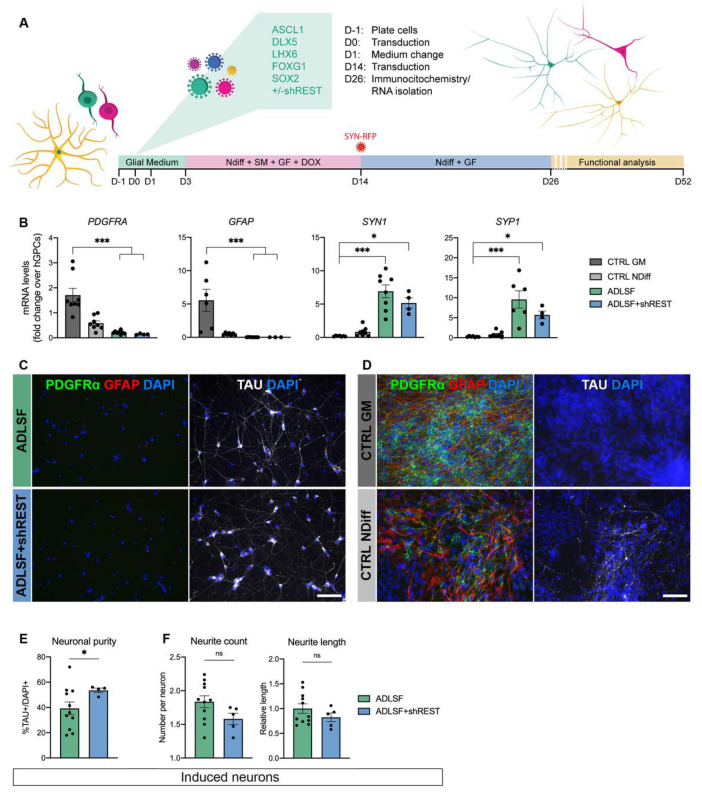
Neuronal conversion of hESC-derived GPCs in 26 days: (**A**) Schematic of the reprogramming strategy using the ADLSF factor combination with or without REST inhibition. (**B**) RT-qPCR analysis showed downregulation of glial markers and upregulation of neuronal genes at day 26 after transduction. Kruskal–Wallis test, Dunn’s multiple comparisons test (n = 6–8 for CTRL GM, n = 7–8 for CTRL NDiff, n = 6–8 for ADLSF, and n = 3–4 for ADLSF + shREST): *** *p* < 0.005; * *p* < 0.05. (**C**,**D**) PDGFRα/GFAP and TAU immunostainings of (**C**) reprogrammed neurons generated with ADLSF and ADLSF + shREST and (**D**) control glial cells kept in glial medium (CTRL GM) or neuronal conversion medium (CTRL NDiff). (**E**) Quantification of immunodetected TAU^+^ cells. Two-tailed unpaired t-test with Welch´s correction (n = 11 for ADLSF and n = 4 for ADLSF + shREST): * *p* = 0.002; df = 11.97. (**F**) Quantification of neurite profile in TAU^+^ induced neurons. No significative difference (ns) was detected when comparing ADLSF (n = 11) and ADLSF + shREST (n = 5) using the two-tailed unpaired t-test. Data are presented as mean ± SEM. Each data point represents a replicate from an independent experiment. For the measurement of neurite length, each datapoint was normalized to the mean of the ADLSF condition. Scale bars: (**C**,**D**) 100 μm. Abbreviations: SM, small molecules; GF, growth factors; DOX, doxycycline.

hESC-derived GPCs were transduced with Ascl1, Dlx5, Lhx6, Sox2, and Foxg1 (hereinafter together referred to as ADLSF) where Sox2 and Foxg1 were de-activated via doxycycline withdrawal after two weeks. The reprogramming cocktail was tested with or without short hairpin (sh) RNAs against the REST complex under a constitutive promotor (Figure 1A). Control cultures of untransduced cells were kept in parallel in either glial medium (GM) or neuronal conversion medium (NDiff, containing small molecules and growth factors).

Twenty-six days after transgene delivery, the hESC-derived GPCs showed an efficient downregulation of the glial marker genes *PDGFRA* and *GFAP* in ADLSF and ADLSF + shREST conditions and a distinct increase in pan-neuronal marker synapsin (*SYN1*) as well as the synaptic vesicle protein synaptophysin (*SYP*), which was significantly higher compared to the controls analyzed in parallel by RT-qPCR (Figure 1B). The neuronal reprogramming was further assessed at protein level using immunocytochemistry. The iN cultures contained few if any PDGFRα^+^ and GFAP^+^ cells, indicating a successful change from the glial fate and instead showed clear expression of the late neuronal marker TAU (Figure 1C). This was in contrast with both control groups that maintained their glial identity (Figure 1D). Quantification of the TAU-expressing neurons in the reprogrammed cultures revealed a 39.2 ± 5.1% neuronal purity in ADLSF and 53.5 ± 1.8% in the ADLSF + shREST group (Figure 1E). Quantitative morphological assessment of TAU^+^ cells using a high-content screening approach showed a similar level of morphological complexity in both reprogramming conditions, but a trend toward higher neurite count and neurite length in ADLSF (Figure 1F).

Together, this indicates that the five-factor combination successfully reprograms hESC-derived GPCs into iNs in 26 days with a high proportion of TAU-expressing cells, especially in the REST knockdown group.

### 3.2. The Induced Cultures Consist of GABAergic Interneurons and Show PV Expression

**Figure 2:**
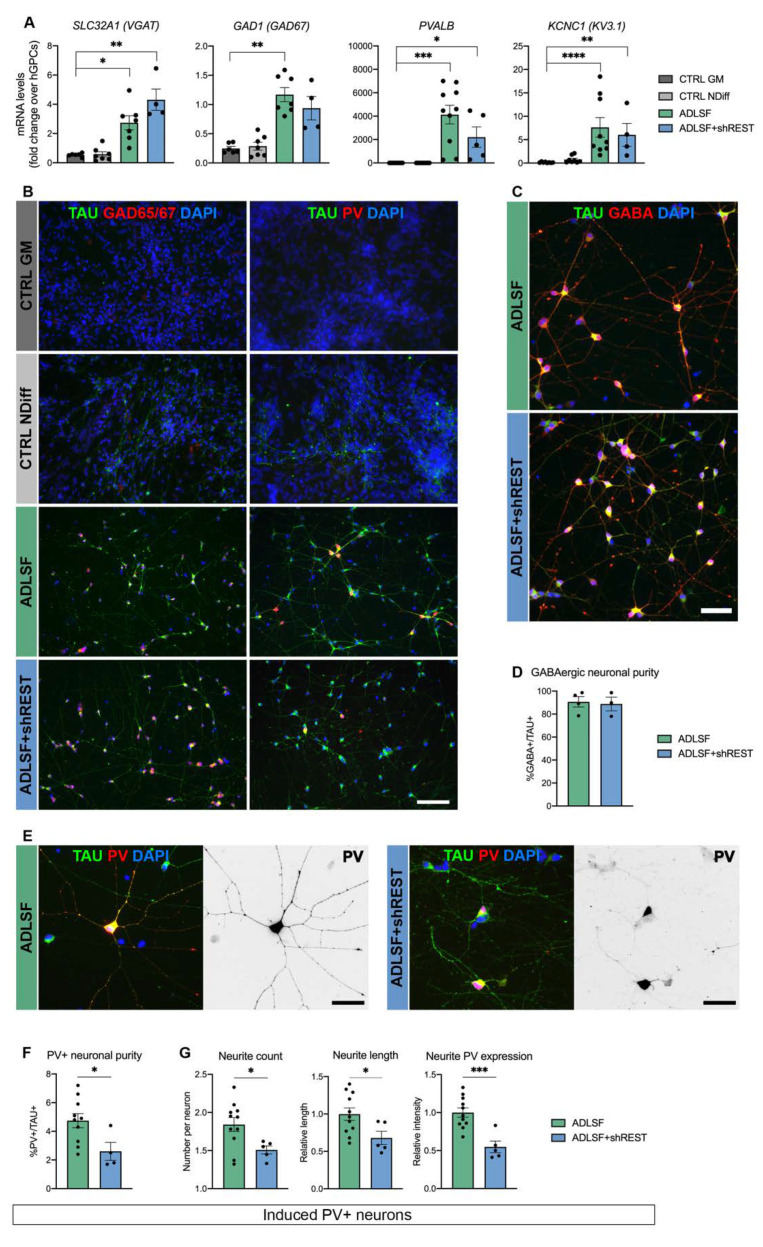
Generation of GABAergic interneurons with PV expression: (**A**) RT-qPCR analysis of GABAergic gene expression at 26 days after transduction. Kruskal–Wallis test, Dunn’s multiple comparisons test (n = 6–10 for CTRL GM, n = 7–9 for CTRL NDiff, n = 7–10 for ADLSF and n = 4–5 for ADLSF + shREST): **** *p* < 0.0001; *** *p* < 0.005; ** *p* < 0.01; * *p* < 0.05. (**B**) Immunocytochemistry of TAU^+^ neurons expressing GAD65/67 and PV in ADLSF w/o shREST at 26 days after transduction. (**C**,**D**) GABA^+^/TAU^+^ cells in reprogrammed cultures, (**C**) immunocytochemistry, and (**D**) quantification. No significative difference was detected between ADLSF (n = 4) and ADLSF + shREST (n = 3) using the two-tailed unpaired t-test. (**E**) Representative image of PV expression in neurites. Fluorescent images are shown in black and white and inverted for clarity. (**F**) Quantification of a higher PV^+^ neuronal proportion in ADLSF condition. Two-tailed unpaired t-test (n = 10 for ADLSF and n = 4 for ADLSF + shREST): *, *p* = 0.032; df = 12. (**G**) Quantification of neurite profile in PV^+^/TAU^+^ neurons. Two-tailed unpaired t-test (n = 11 for ADLSF and n = 5 for ADLSF + shREST): neurite count, * *p* = 0.034; df = 14; neurite length, * *p* = 0.033; df = 14; neurite PV expression, *** *p* = 0.0007; df = 14. Data are presented as mean ± SEM. Each datapoint represents a replicate from an independent experiment. For the measurement of neurite length and PV expression, each datapoint is normalized to the mean of ADLSF condition. Scale bars: (**B**) 100 μm; (**C**,**E**) 50 μm. See also Figure S1.

We next sought to better characterize the identity of the iNs generated with the two reprogramming cocktails. Gene expression analysis demonstrated that ADLSF and ADLSF + shREST efficiently convert the hESC-derived GPCs into GABAergic neurons as revealed by the increased levels of *SLC32A1*, encoding for the vesicular GABA transporter, and *GAD1*, encoding for the glutamic acid decarboxylase GAD67 responsible for the GABA production. This was also accompanied by an upregulation of *PVALB*, encoding for the interneuron calcium-binding protein PV, and *KCNC1*, encoding for voltage-gated potassium-channel subunit responsible for fast-spiking properties, indicating the induction of a PV interneuron phenotype (Figure 2A).

We also evaluated the immunoreactivity for key markers of GABAergic neurons at 26 days after transduction. Consistent with the upregulation in mRNA levels, the reprogrammed TAU^+^ cells were positively stained for GAD65/67 and PV and showed a distinct neuronal morphology (Figure 2B). Our iNs displayed a strong co-localization of GABA and the neuronal marker TAU (Figure 2C), which was quantified as 90.7 ± 4.5% for ADLSF and 88.8 ± 6% for ADLSF + shREST, suggesting a comparably high GABAergic neuronal purity between the groups (Figure 2D). Few GABA^+^ cells were found in the control groups, but co-labeling with the glial marker PDGFRα confirmed that these cells were of a glial identity (Figure S1). The PV^+^ neurons in ADLSF, but not ADLSF + shREST condition, further displayed extensively branched dendritic trees (Figure 2E). Our best yield of PV^+^/TAU^+^ cells was obtained using ADLSF (4.7 ± 0.5%). The addition of shREST to the conversion cocktail resulted in a lower proportion of PV^+^/TAU^+^ cells (2.6 ± 0.6%), suggesting that the REST inhibition did not add any beneficial effect to the generation of PV interneurons (Figure 2F). 

High-content screening demonstrated a less complex neurite morphology of the PV^+^/TAU^+^ iNs generated with ADLSF compared to ADLSF + shREST. In accordance with a reduced yield of PV^+^/TAU^+^ cells, REST knockdown also had a significantly negative impact on the neurite number and length and resulted in a decreased PV expression within the neurites (Figure 2G). 

These results suggest that the five-factor combination without REST inhibition converts hGPCs into a GABAergic-enriched population of iNs with elaborate morphology and some expression of PV. 

### 3.3. Functional Characterization of Induced GABAergic Neurons Obtained in 26 Days

**Figure 3:**
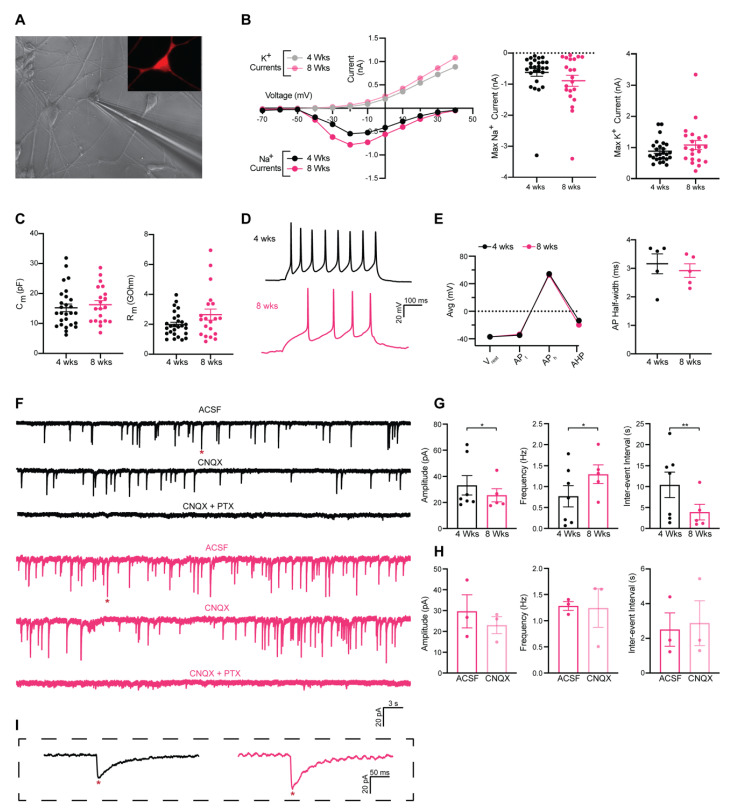
Physiological properties of iNs converted with ADLSF at four weeks and eight weeks. (**A**) Representative image of Syn-RFP^+^ cell in whole cell patch-clamp configuration. (**B**) Inward Na^+^ and outward K^+^ currents plotted against stepwise voltage induction at four weeks (4 wks, black/grey) and eight weeks (8 wks, magenta/pink). Each dot representing the mean value (n = 26 cells, 4 wks and n = 21 cells, 8 wks). (**C**) Measured intrinsic membrane properties, input resistance (left) and membrane capacitance (right). All values are presented as mean ± SEM (n = 27 cells, 4 wks and n = 20 cells, 8 wks). (**D**) Representative trace of repetitive action potentials (APs) evoked by rheobase current injection steps, 4 wks (black) 8 wks (magenta). (**E**) AP properties, resting membrane potential (V_rest_), AP threshold (AP_t_) AP amplitude (AP_h_) and after-hyperpolarization (AHP, left). Each dot representing the mean value (n = 5 cells, 4 wks and n = 5 cells, 8 wks). Half-width of the first evoked AP (right). Values are presented as mean ± SEM (n = 5 cells, 4 wks and n = 5 cells, 8 wks). (**F**) Sample traces of postsynaptic activity blocked with 50 µM CNQX and 50 µM CNQX + 100 µM PTX at 4 wks (black) and 8 wks (magenta). (**G**) Amplitude, frequency, and interevent interval of postsynaptic activity at 4 wks and 8 wks. Values are mean ± SEM (n = 7 cells, 4 wks and n = 5 cells, 8 wks). (**H**) Amplitude, frequency, and interevent interval of postsynaptic activity at 8 wks ACSF and ACSF + 50 µM CNQX. Values are presented as mean ± SEM (n = 3 cells). (**I**) Magnification of representative inhibitory postsynaptic events (red asterisk) at four weeks (black) and eight weeks (magenta). Two-tailed, unpaired Mann–Whitney (** *p* < 0.01; * *p* < 0.05) was used for all comparisons except postsynaptic activity with antagonist (CNQX), where paired Wilcoxon rank-sum tests were performed. See also Figure S2.

In the next set of experiments, we examined whether hESC-derived GPC transduced with neurogenic fate determinants, also acquired the electrophysiological hallmarks of neurons such as the ability to fire action potentials (AP). To this end, we performed patch-clamp recordings of hESC-derived GPCs transduced with ADLSF. It has previously been reported that human interneuron progenitors follow a prolonged intrinsic timeline for maturation [33], therefore whole-cell recordings were done at four weeks and eight weeks after conversion to evaluate a potential maturation in the electrical functions. One week prior to recording cells were transduced with hSyn-RFP for the visualization and selection of successfully converted neurons (Figure 3A).

To assess if the iNs matured over time, we compared their voltage-gated sodium (Na^+^) and potassium (K^+^) currents and intrinsic membrane properties at four weeks and eight weeks. The iNs displayed both inward and outward rectifying Na^+^ and K^+^ currents with no significant change between the time points (Figure 3B). Both Na^+^ and K^+^ currents could be blocked with the antagonists tetrodotoxin (TTX) and tetraethyl ammonium (TEA), respectively. For input resistance and cell capacitance, the values varied greatly within the groups (Figure 3C), suggesting that the pace of maturation is highly heterogenous. Thus, individual iNs appear to develop quicker than others. In line with this, some cells showed the ability to fire repetitive series of evoked AP upon current injection already at four weeks (5/27), indicative of neuronal function (Figure 3D). There was a similar proportion of firing neurons at eight weeks (5/21). The discharge pattern and AP generation properties such as resting membrane potential, AP threshold, AP amplitude, and afterhyperpolarization (AHP) remained similar between the time points, suggesting that cells already achieved maturity at four weeks and did not develop further (Figure 3E). APs were fully blocked with TTX, demonstrating their Na-channel dependence (Figure S2).

We next assessed the ability of the reprogrammed cells to form synapses. At four weeks post-transfection, a large part of iNs showed an absence of spontaneous synaptic activity, however, a proportion of iNs (6/27) showed the presence of postsynaptic activity, suggesting the formation of a functional network. At eight weeks, there was a similar proportion of cells showing synaptic activity (5/21) with an increased frequency and inter-event interval (Figure 3F,G), indicating more synaptic connectivity in the iN cultures. 

In line with our gene and protein analysis, the postsynaptic events showed the typical shape of GABAergic inhibitory postsynaptic current (iPSC) events with a long decay time (>20 ms) (Figure 3I). Whereas the AMPA antagonist cyanquixaline (CNQX) did not produce any effect on the amplitude, frequency, or inter-event interval (Figure 3H), the GABA_A_ receptor (GABA_A_R) antagonist picrotoxin (PTX) completely blocked the spontaneous events (Figure 3F). This demonstrates that the cultures are comprised of GABA_A_R mediated synapses, which is consistent with the vast majority of neurons being GABAergic.

In conclusion, these data suggest that hESC-derived GPCs can be converted into functional iNs that within four weeks are capable of producing evoked AP and forming a synaptic network that is of GABAergic nature.

### 3.4. A Heterogenous Population of GABAergic Induced Neurons

**Figure 4:**
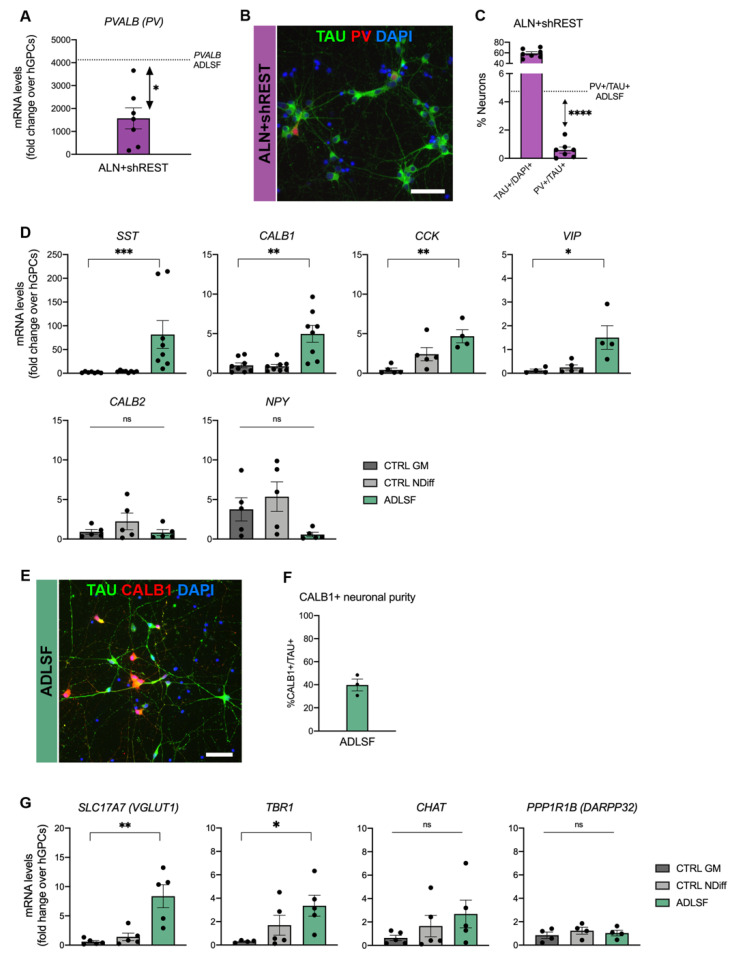
iNs display molecular properties of interneurons at day 26 after transduction. (**A**) RT-qPCR analysis of *PVALB* gene expression in ALN + shREST reprogrammed neurons and comparison with ADLSF. Two-tailed unpaired *t*-test with Welch´s correction (n = 7 for ALN + shREST): * *p* = 0.025; df = 15; dashed line represents mean average of *PVALB* mRNA levels in ADLSF condition (see Figure 2A). (**B**,**C**) TAU^+^ and PV^+^/TAU^+^ cells in ALN + shREST reprogrammed neurons, (**B**) immunocytochemistry, and (**C**) quantification with comparison to ADLSF. Two-tailed unpaired *t*-test with Welch´s correction (n = 7 for ALN + shREST): **** *p* < 0.0001; df = 12.07; dashed line represents mean average of PV^+^/TAU^+^ cells in ADLSF condition (see Figure 2F). (**D**) RT-qPCR analysis of ADLSF reprogrammed cells showing upregulation for subtype-specific GABAergic interneuron markers. Kruskal-Wallis test, Dunn’s multiple comparisons test (n = 4–8 for CTRL GM, n = 5–8 for CTRL NDiff, n = 4–8 for ADLSF): *** *p* < 0.005; ** *p* 0.01; * *p* < 0.05; ns, not significant. (**E**,**F**) Reprogrammed CALB1^+^/TAU^+^ (n = 3) at 26 days post transduction, (**E**) immunocytochemistry, and (**F**) quantification. (**G**) RT-qPCR analysis of different neuronal lineage markers in ADLSF-reprogrammed cells. Kruskal–Wallis test, Dunn’s multiple comparisons test (n = 4–5 for CTRL GM, for CTRL NDiff and ADLSF): ** *p* < 0.01; * *p* < 0.005; ns, not significant. Data are presented as mean ± SEM. Each datapoint represents a replicate from an independent experiment. Scale bar: (**B**,**E**) 50 μm.

Previous data from our lab have demonstrated that PV interneurons can be generated by direct in vivo reprogramming of mouse GPCs using the transcription factors Ascl1, Lmx1a, and Nurr1 (together referred as to ALN) [17], whereas this combination with or without REST knockdown gives rise to DA neurons when fibroblasts or glia are converted in vitro [28,34,35]. Here, we evaluated if ALN + shREST could also convert hGPCs into PV^+^ GABAergic interneurons and improve the PV yield compared to ADLSF. Even though iNs reprogrammed with ALN + shREST showed an upregulation in the mRNA levels for PVALB at 26 days post transduction (Figure 4A), only 0.6 ± 0.2% of the iNs expressed PV protein (out of 59 ± 3.1% TAU^+^/DAPI^+^ cells; Figure 4B,C), which was significantly less than the ADLSF condition. This demonstrates that the ADLSF combination is superior to ALN + shREST in directing the fate from glia toward PV interneurons in vitro.

However, a fair proportion of the ADLSF-reprogrammed neurons remained PV negative and therefore we next investigated the expression of markers specific for other GABAergic interneuron subtypes in the iN cultures. In addition to upregulation of *PVALB*, the iNs also showed significantly increased mRNA levels of somatostatin (*SST*) as well as calbindin (*CALB1*), cholecystokinin (*CCK*), and vasoactive intestinal polypeptide (*VIP*). Conversely there was no upregulation of calretinin (*CALB2*) and neuropeptide Y (*NPY*) (Figure 4D). This gene expression analysis suggests a successful conversion into distinct interneuron populations. CALB1 could also be detected at the protein level (Figure 4E) and quantified as 39.8 ± 5.1% of the TAU^+^ iNs (Figure 4F). 

Additional RT-qPCR analysis for other neuronal linage markers revealed an increased gene expression of the glutamatergic marker *SLC17A7 (VGLUT1)* and *TBR1*, a cortical marker of postmitotic projection neurons [36], and a neglectable *CHAT* expression, marker of cholinergic neuronal subtypes. There was no significant gene expression of *PPP1R1B* encoding for the medium spiny neuron marker *DARPP32* (Figure 4G), supporting a GABAergic interneuron fate instead of a striatal projection neuron fate.

Together, these data suggest that the induced cultures generated from hESC-derived GPCs contain a heterogenous spectrum of GABAergic interneuron subtypes and, to a smaller extent, some glutamatergic neurons. 

## 4. Discussion

Neural reprogramming of endogenous glia directly within the brain parenchyma represents an appealing alternative approach for cell-replacement therapy of neurological disorders that avoids cell transplantation and its associated risks including the need for immunosuppression [6]. In particular, direct conversion into GABAergic interneurons could address the altered neuronal circuitry in a range of neurological and neuropsychiatric conditions produced by the hypofunction/degeneration of these neurons [21,22]. While a number of studies including our own has already highlighted the potential of mouse GPCs (NG2 cells) as a candidate for the in vivo generation of GABAergic neurons and interneurons [14,17,37], analogous studies using human cells have been lacking. Thus far, reprogrammed human fetal astrocytes mainly adopt a glutamatergic phenotype [12,38,39] and can yield low [38,39] or some [39] GABAergic neurons when from the cortical or midbrain origin, respectively, while GABAergic neurons have instead been obtained from adult human brain pericytes [40]. Nevertheless, GPCs might hold further advantages over both astrocytes and pericytes such as proliferative capacity, wide distribution, and an already established neuronal network [8,41,42]. 

We have recently developed a stem cell-based system that can model hGPC-conversion in vitro [25,28]. In this study, we used these hESC-derived GPCs for neuronal conversion with a focus on GABAergic interneurons. To this end we applied Ascl1, Dlx5, and Lhx6, well-known inducers of GABAergic interneuron fate [43,44,45], in combination with the timed delivery of Sox2 and Foxg1, recapitulating the development of forebrain interneurons in vivo [46,47]. Thereof, Sox2 and Foxg1 expression was turned off after the first 12–14 days of reprogramming to allow a correct maturation of the iNs [26].

Our results demonstrate a successful reprogramming of hGPCs into a GABAergic-enriched population of neurons expressing genes characteristic of GABAergic interneurons and displaying inhibitory postsynaptic activity within 26 days, which is significantly faster compared to the extended timeline required by differentiation protocols relying on human pluripotent stem cells (hPSCs) [33,48]. The GABAergic yield was substantially higher than previous in vitro conversions of human fetal astrocytes using a different chemical reprogramming approach [38,39]. One of the reasons for this high GABAergic purity could be that GPCs already express some GABAergic components [10] and potentially synthetize GABA [49], findings that thus far has only been shown in rodents. In this study, the co-localization of PDGFRα and GABA could also be detected in a few hGPCs. Importantly, GPCs also share the developmental origin with cortical GABAergic interneurons deriving from the medial ganglionic eminence (MGE) [50,51], which may contribute to their GABAergic properties after reprogramming.

The induced GABAergic neurons also showed PV immunoreactivity in cells with a remarkably extensive mature neuronal morphology. Endogenous PV interneurons represent a third of the interneuron population in the neocortex, are divided into basket or chandelier cells [52] and arise from the MGE along with the SST interneurons. PV cells are key cellular elements in controlling excitability in the brain and deficits of these are thought to be a main underlying mechanism behind pathological conditions such as epilepsy, schizophrenia, and anxiety [21,53]. Despite a major clinical interest in these neurons and efforts to obtain them from ESCs [48], the efficient generation of mature PV interneurons have proven to be challenging [54] and thus far, only been shown in a few fibroblast-based [26] and hPSC-based conversion experiments [55,56]. Here, we provide the first evidence of the successful generation of this type of interneuron also from hGPCs. The reprogrammed cells displayed molecular properties of SST interneuron subtypes as well as other cortical interneuron subpopulations derived from caudal ganglionic eminences indicated by the upregulation of *CCK* and *VIP* mRNA levels [52] and included CALB1-immunopositive cells. The small non-GABAergic population could be addressed by the expression of glutaminergic and projection neuron molecular markers; however, the nature of these cells remains to be characterized. Additional work is needed to fully investigate if a different reprogramming cocktail could increase the yield of PV interneurons.

In our reprogramming protocol, we also investigated the role of REST, a transcriptional repressor known to silence neuronal genes in stem cells and non-neural cells [57,58] including both GPCs and differentiating astrocytes [59,60]. Our results confirm an increase of TAU expression when inhibiting REST, but at the expense of a lower PV expression and less extensive neuronal morphology. This might be explained by the broad effects of REST on neuronal identity and function by controlling a large cohort of positively and negatively interacting epigenetic modulators, transcription factors, and neuronal differentiation genes [61]. A more detailed understanding of REST function in the context of different neuronal subtypes would be necessary to further elucidate the potential of its inhibition for conversion purposes, but this was outside the scope of this study.

Electrophysiological recordings confirmed that the converted hGPC cultures are highly GABAergic with GABAergic postsynaptic activity detected after four weeks. This is comparable to other protocols of stem cell-derived GABAergic neurons [62]. While the presence of increased synaptic activity between four and eight weeks could indicate that more cells are integrated in the postsynaptic network or mature in culture over time, individual cells appeared similar in firing patterns at both time points. However, despite upregulation of *KCNC1* gene expression suggesting the presence of neurons with fast-spiking properties, we could not identify any cells with high-frequency patterns in our cultures. Yet, given the relatively low proportion of PV^+^ neurons (and the lack of a PV reporter), our assessment showed the functional properties of the general GABAergic population of iNs. One can still speculate that cells have not yet reached full maturity, which could require a prolonged culture condition to develop this firing pattern. Another explanation could be the homogeneous environment of the cells in culture, as the in vivo counterpart is known to require an activity-dependent mechanism of principal cells and other interneurons to both reach maturity and integrate properly [19]. In the future, alternative culture systems should be explored to potentially enhance long-term physiological maturation of converted cells (e.g. co-cultures with cortical cells), which has been shown to be a prominent method for human MGE-like cells [48]. Ultimately, the hGPC-derived interneurons should be transplanted into a disease-relevant part of the brain to unravel the true functional maturation as well as the potential clinical relevance of these neurons [33,63].

In conclusion, uncovering the possibility of generating functional GABAergic neurons from our hESC-based reproducible GPC system contributes to the characterization of GPCs as a promising target for in vivo direct conversion approaches aimed at restoring the interneuron deficits of the diseased brain.

## Supplementary Materials

The following are available online at https://www.mdpi.com/2073-4409/9/11/2451/s1, Table S1: Primers used for RT-qPCR analysis, Table S2: List of primary antibodies used in this study, Table S3: List of cell batches used as starting cells for reprogramming experiments, Figure S1: Immunocytochemistry of GABA and glial markers in control groups after 26 days in culture, Figure S2: Inward Na+ and outward K+ currents blocked by TTX+TEA at 8 weeks.
