# Supplementary material for: Direct Conversion of Human Stem Cell-Derived Glial Progenitor Cells into GABAergic Interneurons
Source: Cells. 2020 Nov 10;9(11):2451. doi: 10.3390/cells9112451 (PMC7698048; doi:10.3390/cells9112451)
Supplement: Supplementary file 1 [file cells-09-02451-s001.zip › Giacomoni et al._Supplementary files/Giacomoni et al._Supplementary Materials.docx]

***SUPPLEMENTARY MATERIALS***

**Table S1.** Primers used for RT-qPCR analysis.

| **Gene** | **Full gene name** | **Primer sequence (fwd/rev)** |
| --- | --- | --- |
| *ACTB* | Beta-actin | CCTTGCACATGCCGGAG  CCTTGCACATGCCGGAG |
| *CALB1* | Calbindin | TGGCTCCATTTCGACGCTGACG  ATCCAGCCTTCTTTCGCGCCTG |
| *CALB2* | Calretinin | TGGAGGCTTGGCGGAAGTACGA  CCGGTTCGCCTTCTTCAGCAGG |
| *CCK* | Cholecystokinin | AGGGTATCGCAGAGAACGGA  CTTATCCTGTGGCTGGGGTC |
| *CHAT* | Choline O-acetyltransferase | TCCAACGAGGACGAGCGTTTGC  CGAGTCCCGGTTGGTGGAGTCT |
| *PPP1R1B (DARPP32)* | Dopamine- and cAMP-regulated neuronal phosphoprotein | GAGAGCCTCAGGAGAGGGGCAC  AGGTGGTGTGTAGGCACAGGGG |
| *GAD1 (GAD67)* | Glutamate decarboxylase 1 | CTCCTGGGGGCGCCATATCCAA  CCAGTTTAGGCACAGCCGCCAT |
| *GAPDH* | Glyceraldehyde-3-phosphate dehydrogenase | TTGAGGTCAATGAAGGGGTC  GAAGGTGAAGGTCGGAGTCA |
| *GFAP* | Glial fibrillary acidic protein | TCATCGCTCAGGAGGTCCTT  CTGTTGCCAGAGA TGGAGGTT |
| *NPY* | Pro-neuropeptide Y | TGTTCCCAGAACTCGGCTTG  TGCATTGGTAGGATGGGTGG |
| *PDGFRA* | Platelet-derived growth factor receptor alpha | CCTTGGTGGCACCCCTTAC  TCCGGTACCCACTCTTGATCTT |
| *PVALB* | Plasmalemma vesicle-associated protein | TGCAGGATGTCGATGACAGA  TTTCTTCAGGCCGACCATTT |
| *SST* | Somatostatin | CAAGCCGCTTTAGGAGCGAG  AGGCGGCAGGACAGCATCT |
| *SYN1* | Synapsin 1 | CCCGTGGTTGTGAAGA TGGGGC  TGCCACGACACTTGCGATGTCC |
| *SYP* | Synaptophysin | ACCTCGGGACTCAACACCTCGG  GAACCACAGGTTGCCGACCCAG |
| *TBR1* | T-box brain protein 1 | TCGTCCCCGCTCAAGAGCGA  CCTTGGCGCAGTTCTTCTCGCA |
| *SLC32A1 (VGAT)* | Vesicular inhibitory amino acid transporter | AGATGATGAGAAACAACCCCAG  CACGACAAGCCCAAAATCAC |
| *SLC17A7 (VGLUT1)* | Vesicular glutamate transporter 1 | AATAACAGCACGACCCACCGCG  AGCCGTGTATGAGGCCGACAGT |
| *VIP* | Vasoactive intestinal peptide | TCTCACAGACTTCGGCATGG  TCATTTGCTCCCTCAAAGGGT |
| *KCNC1 (KV3.1)* | Potassium voltage-gated channel subfamily C member 1 | GCTGTTCGAGGACCCCTACT  GTTGAAGCGCTCGTGAGTCT |

**Table S2.** List of primary antibodies used in this study.

| **Marker** | **Specificity** | **Source (cat. no.)** | **Dilution** |
| --- | --- | --- | --- |
| CALB1 | Rabbit | Swant (CB38) | 1:500 |
| GABA | Rabbit | Sigma (A2052) | 1:2000 |
| GAD65/67 | Rabbit | Abcam (AB49832) | 1:2000 |
| GFAP | Mouse | Biolegend (SMI 21) | 1:500 |
| PDGFRα | Rabbit | Cell Signaling Technology (5241S) | 1:300 |
| PDGFRα | Goat | R&D Systems (AF-307-NA) | 1:500 |
| PV | Rabbit | Swant (PV27) | 1:500 |
| PV | Mouse | Sigma (P3088) | 1:2000 |
| TAU (HT7) | Mouse | Thermo Fisher (MN1000) | 1:500 |

**Table S3.** List of cell batches used as starting cells for reprogramming experiments.

| **hESC line** | **hGPC Batch ID** | **Days in culture** | **Total CD140+** | **Total CD44+** | **CD140+/**  **CD44-** | **CD140+/**  **CD44+** | **CD140-/**  **CD44+** |
| --- | --- | --- | --- | --- | --- | --- | --- |
| RC17 | LU24 | 304 | 60.8 % | 34.5 % | 33.1 % | 24.6 % | 0.88 % |
| RC17 | J2 | 228 | 68.8 % | 27.6 % | 44.3 % | 26.0 % | 0.65 % |
| RC17 | J4 | 176 | 71.3 % | 17.7 % | 50.3 % | 19.6 % | 1.08 % |
| RC17 | LU19 | 210 | 54.0 % | 24.7 % | 29.6 % | 18.7 % | 0.41 % |
| HS1001 | LU29 | 215 | 53.10% | 30.6 % | 24.7 % | 28.3 % | 1.53 % |
| RC17 | LU13 | 240 | 53.7 % | 20.5 % | 35.7 % | 15.0 % | 0.34 % |
| RC17 | LU28 | 233 | 46.7 % | 5.09 % | 42.9 % | 3.32 % | 0.59 % |
| RC17 | J4 | 214 | 59.3 % | 6.54 % | 53.2 % | 4.94 % | 0.35 % |
| RC17 | LU6 | 275 | 55.5 % | 7.26 % | 47.5 % | 7.11 % | 0.22 % |
| RC17 | JM001 | 188 | 77.9 % | 25.4 % | 58.3 % | 14.6 % | 2.08 % |
| RC17 | LU7 | 270 | 56.8 % | 8.94 % | 47.3 % | 8.96 % | 0.53 % |
| RC17 | LU13 | 211 | 47.9 % | 6.06 % | 41.4 % | 6.35 % | 0.21 % |


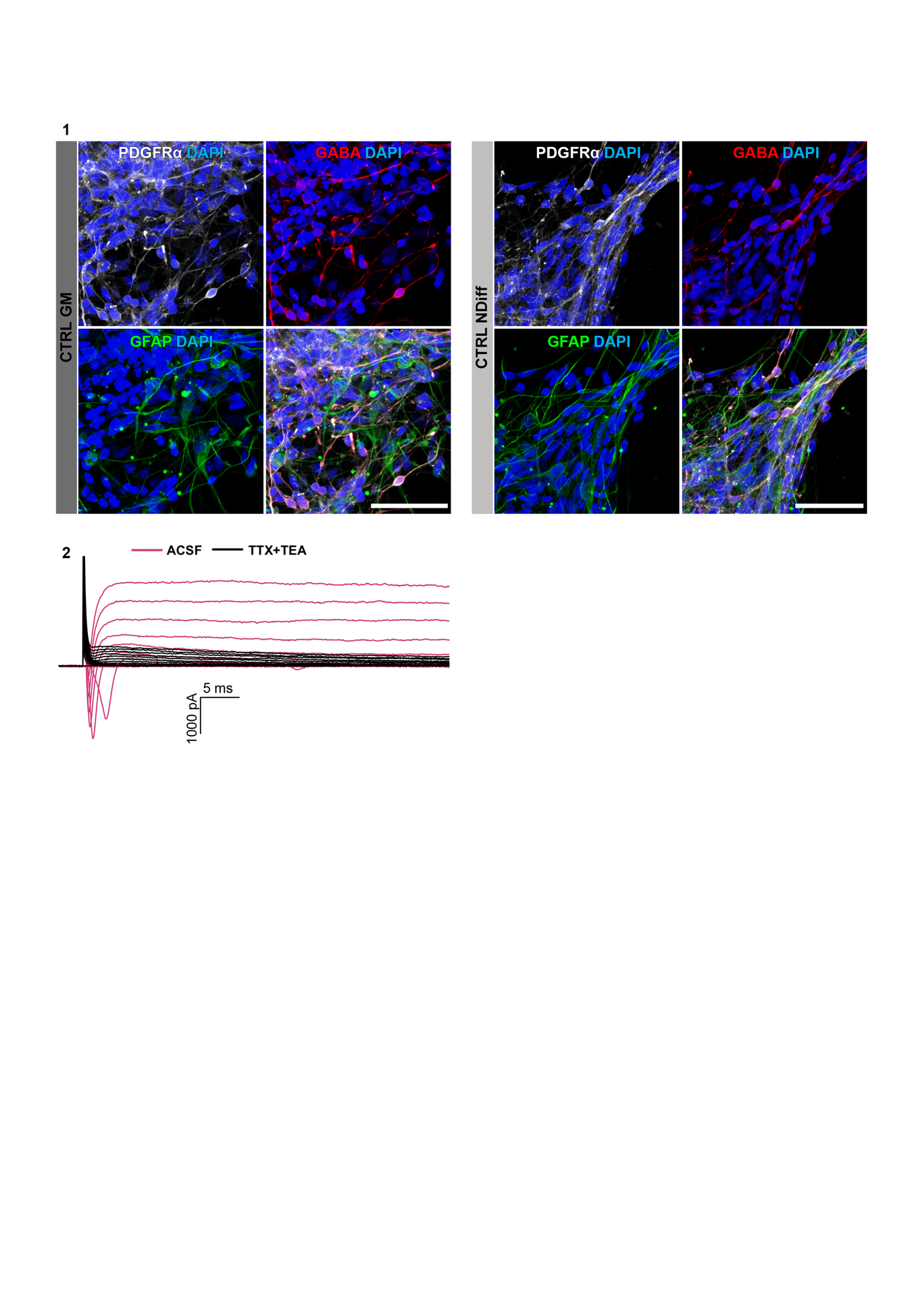


**Figure S:** (**1**) Immunocytochemistry of GABA and glial markers in control groups after 26 days in culture. Scale bars: 50 µm. (**2**) Inward Na^+^ and outward K^+^ currents blocked by TTX+TEA at 8 weeks.
